# Typical 22q11.2 deletion syndrome appears to confer a reduced risk of schwannoma

**DOI:** 10.1038/s41436-021-01175-0

**Published:** 2021-04-20

**Authors:** D. Gareth Evans, Ludwine M. Messiaen, William D. Foulkes, Rachel E. A. Irving, Alexandra J. Murray, Cristina Perez-Becerril, Barbara Rivera, Donna M. McDonald-McGinn, David A. Stevenson, Miriam J. Smith

**Affiliations:** 1 Manchester Centre for Genomic Medicine, St Mary’s Hospital, Manchester Academic Health Science Centre, Division of Evolution and Genomic Science, School of Biological Sciences, University of Manchester, Manchester, UK; 2 Medical Genomics Laboratory Department of Genetics, University of Alabama at Birmingham, Birmingham, AL USA; 3 Department of Human Genetics, McGill University, Montreal, QC Canada; 4 All Wales Medical Genomics Service, University Hospital of Wales, Heath Park, Cardiff, UK; 5 Program in Molecular Mechanisms and Experimental Therapy in Oncology, IDIBELL, Hospitalet de Llobregat, Barcelona, Spain; 6 Division of Human Genetics and 22q and You Center, Children’s Hospital of Philadelphia, Philadelphia, PA USA; 7 Department of Pediatrics, Perelman School of Medicine of the University of Pennsylvania, Philadelphia, PA USA; 8 Division of Medical Genetics, Stanford University, Stanford, CA USA

## Abstract

**Purpose:**

The *LZTR1* gene has been associated with schwannomatosis tumor predisposition and is located in a region that is deleted in the great majority (89%) of patients with 22q11.2 deletion syndrome (22q11.2DS). Since it is known that approximately 1 in 500 people in the general population will develop a sporadic schwannoma and there are no reports of the occurrence of schwannoma in 22q11.2DS, we investigated whether whole-gene deletion of *LZTR1* occurs in schwannomatosis and assessed the risk of schwannoma in 22q11.2DS.

**Methods:**

We assessed the genetic testing results for *LZTR1*-associated schwannomatosis and the clinical phenotypes of patients with 22q11.2DS.

**Results:**

There were no reports of schwannoma in over 1,500 patients with 22q11.2DS. In addition, no patients meeting clinical diagnostic criteria for schwannomatosis had a whole-gene deletion in *LZTR1*. Only 1 patient in 110 with an apparently sporadic vestibular schwannoma had a constitutional whole-gene deletion of *LZTR1*.

**Conclusion:**

People with a large 22q11.2 deletion may have a reduced risk of developing a schwannoma compared to the general population.

## Graphical abstract

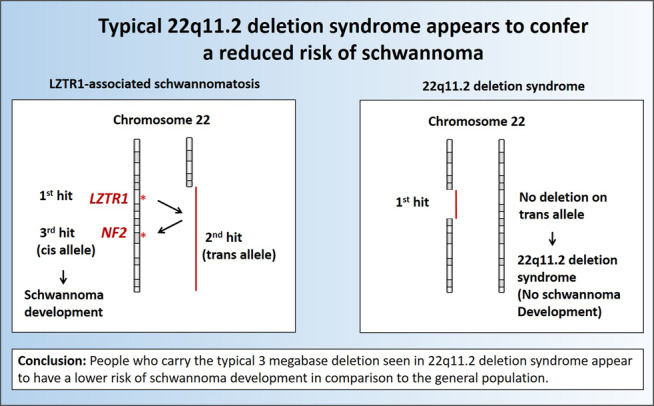

## INTRODUCTION

Heterozygous loss-of-function pathogenic variants in *LZTR1* are a known cause of schwannoma predisposition^1^ and account for around 30% of both inherited and sporadic cases of schwannomatosis.^2^ The mechanism of schwannoma formation resulting from a germline pathogenic variant in *LZTR1* on chromosome 22 is more complex than the traditional two-hit hypothesis^3^ and is thought to require biallelic loss of *NF2* as well as *LZTR1.*^4,5^ Following the predisposing germline event (hit 1), a subsequent, acquired loss of the unaffected copy of 22q deletes the remaining copy of *LZTR1* and also one copy of *NF2* (a second event including two genetic hits). The final (third) event is the additional acquisition of a loss-of-function variant (usually of a single nucleotide) in *NF2*.^5^ This three-event, four-hit mechanism is now well established as the cause of schwannomas both through the *SMARCB1* and *LZTR1* pathways. Whole-gene deletion and other copy-number variants in *SMARCB1* are typically linked to rhabdoid tumor predisposition syndrome (RTPS) and have not been reported to cause schwannomas.^6^ Typically, schwannomatosis-associated *SMARCB1* variants that cause around 40% of hereditary and 10% of sporadic schwannomatosis are “hypomorphic” with some presumed preserved function of the affected allele.^6^ This preserved function appears to be sufficient to prevent development of rhabdoid tumors in the critical first few years of life. Typical *LZTR1* variants that cause schwannomatosis are more likely to be truncating variants, such as frameshift deletions and nonsense variants that are predicted to lead to complete loss of protein, while nontruncating variants are less common.^2^ However, whole-gene deletions have not been reported in schwannomatosis.

The *LZTR1* gene is located within chromosome 22q11.2. This region is known to be deleted in the majority of people with 22q11.2 deletion syndrome (22q11.2DS), which occurs in approximately 1 in 3–6,000 live births.^7^ The 22q11.2 deletions occur between four low copy repeat regions, LCR22A, LCR22B, LCR22C, and LCR22D, varying in size from approximately 700 kilobases to three megabases. The *LZTR1* gene is located within the LCR22C–LCR22D region. The most common deletion is three megabases, caused by nonallelic homologous recombination (NAHR) between LCR22A and LCR22D. The major features of 22q11.2DS are immunodeficiency, palatal anomalies, hypoparathyroidism, and congenital heart disease (CHD). There are many other possible associated features of this condition, such as skeletal, renal, and gastrointestinal differences, autoimmune disease, cognitive deficits, behavioral differences, and psychiatric illness. However, schwannomas have not been reported in people with this deletion and whole-gene deletions have not been reported in *LZTR1*-associated schwannomatosis. We sought to investigate the risk of schwannomas in 22q11.2 deletion syndrome and the association with whole-gene deletion in *LZTR1*-associated schwannomatosis.

## MATERIALS AND METHODS

Two large genetic testing databases were interrogated to identify *LZTR1* variants in individuals who presented with schwannomas. A total of 247 individuals were identified with an *LZTR1* variant. Of these, 124 were identified through the Manchester Centre for Genomic Medicine and 123 were identified through the genetic testing service at Birmingham, Alabama. This excluded class 3 variants with low likelihood of reclassification to likely pathogenic. These samples were analyzed using a combination of Sanger sequencing, and in-house next-generation panel sequencing (including the *NF2*, *SMARCB1*, and *LZTR1* genes). Copy number was assessed by multiple ligation-dependent probe amplification (MLPA), using probe sets P043-NF2, P258-B1, and P455 LZTR1, respectively (MRC-Holland, Amsterdam, the Netherlands), or by microarray analysis using the OGT CytoSure Constitutional v3 8x60k array or Illumina Infinium CytoSNP-850K array.

To determine the number of schwannomas found in people with 22q11.2 deletion syndrome, 1,556 patients with known 22q11.2 deletion syndrome were identified through the 22q and You Center at the Children’s Hospital of Philadelphia. Loss of 22q11.2 was assessed in the majority by microarray analysis and hospital records were assessed for associated conditions. Of these patients, 322 were assessed by clinical brain magnetic resonance image (MRI) due to a history of seizures/abnormal neurologic exam and another 94 were assessed by brain MRI as part of a research study. In addition, 94 were assessed by spine MRI due to a suspicion of a tethered cord. Furthermore, a survey of the UK database of 22q11.2DS self-reported cases was conducted through social media and by email through the Max Appeal charity.

## RESULTS

**Fig. 1:**
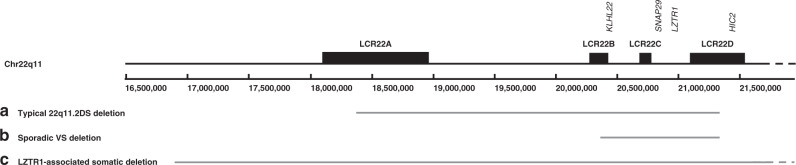
Schematic diagram of chr 22q11.2 indicating low copy repeat regions. Solid gray bars indicate (**a**) the common 3-mb deletion region seen in 22q11.2DS (upper bar), (**b**) the deleted region in the *LZTR1*-associated vestibular schwannoma patient with a whole-gene deletion (middle bar), and (**c**) the approximate breakpoints in tumors from two unrelated *LZTR1*-associated schwannomatosis patients (lower bar).

We reviewed the genetic testing results for schwannoma predisposition in over 1,000 cases from unrelated families in two large genomic testing laboratories. In a total of 247 identified *LZTR1* variants, there were no single or multiple exon deletions, and no whole-gene deletions, identified in patients fulfilling schwannomatosis criteria. A denominator for schwannomatosis was not known in Birmingham, Alabama. However, 116/425 (27.3%) of those meeting schwannomatosis criteria in Manchester (100/400 unrelated families [25%]) had a variant in *LZTR1* (excluding class 3 variants of uncertain significance). A further 8 individuals with an isolated schwannoma in Manchester had an *LZTR1* variant. This included 4/110 patients who presented with sporadic vestibular schwannoma who were screened for *LZTR1* variants. In this group, one of the four *LZTR1* pathogenic variant positive cases had a germline whole-gene deletion. Microarray analysis indicated that the deletion extended to 942 kilobases, between LCR22B-D domains (Fig. 1). The patient, who was in her early 20s, had no features of 22q11.2DS. She had not had her tumor removed, so further investigation of the mechanism of schwannoma formation was not possible (it is normal procedure to watch and wait and rescan a small vestibular schwannoma as they may not need surgery or other treatment for many years).

Age of onset of first schwannoma was available on 122 patients (range 3–78) and confirmed a median of 38 years (mean = 39.6) with 24 aged <29 years.

We next assessed the known incidence of schwannoma in 22q11.2 deletion syndrome. It is known that the typical three megabase deletion that accounts for 85% of cases includes complete deletion of *LZTR1*.^8^ Yet no single case of schwannoma appears in 3,054 reports of Di George syndrome on PubMed (searches using the terms: Di George syndrome AND “schwannoma” and 22q11.2 deletion syndrome AND “schwannoma” were carried out at https://pubmed.ncbi.nlm.nih.gov/?term=Di+George+syndrome+&sort=date, 22 September 2020), nor in 3,031 reports of 22q11.2 deletion syndrome. We also assessed known incidence in over 1,556 patients with known 22q11.2 deletion syndrome evaluated in the 22q and You Center at the Children’s Hospital of Philadelphia. Of those with microarray analysis, 1,046/1,151 (89%) patients had a deletion involving *LZTR1* (Table 1). A further 405 individuals had only fluorescence in situ hybridization (FISH) analysis. No cases of schwannoma have been observed in this cohort, despite over 51% of these patients being adults. In addition, a total of 418 of these patients were assessed by brain MRI and 94 were assessed by spine MRI and no schwannomas were detected.

**Table 1:** 22q11.2 cases from the 22q and You Center at the Children’s Hospital of Philadelphia registry and from the UK charity Max Appeal.

Patient age (years)	*LZTR1*-deleted			*LZTR1* nondeleted			FISH/unknown			UK Max Appeal^a^	
	*n*	% (All)	% (Known)	*n*	% (All)	% (Known)	*n*	% (All)	% (Known)	*n*	% (All)
0 to 18	545	72%	88%	75	10%	12%	132	18%	–	1,013	60%
19 to 29	298	59%	91%	31	6%	9%	175	35%	–	292	17%
30 to 49	134	65%	94%	9	4%	6%	64	31%	–	220	13%
50+	24	67%	86%	4	11%	14%	8	22%	–	170	10%
Deceased/unknown	25	44%	81%	6	11%	19%	26	46%	–		
Total	1,026	66%	89%	125	8%	11%	405	26%	–	1,695	

*FISH* fluorescence in situ hybridization.

^a^UK Max Appeal cases where self-reported and presence or absence of deletion of *LZTR1* was unknown. Furthermore, no record assessment or confirmation of the 22q11.2 deletion was possible. Survey was carried out by Twitter, Facebook, and email between 1 and 30 October 2020.

A survey of 1,695 UK self-reported individuals with 22q11.2DS and their families through the family support organization, Max Appeal, did not identify any cases of schwannoma or nerve sheath tumor, although several likely schwannomas were identified in siblings and parents without known 22q11.2DS.

We also assessed the rate of probable *LZTR1* loss-of-function variants in gnomAD data (https://gnomad.broadinstitute.org/transcript/ENST00000215739?dataset=gnomad_r2_1; 20 January 2021). There were 465 from a mean of 112,096 individuals (~1 in 241) with a nonsense, frameshift, or canonical splice region variant (excluding low confidence calls). This means that if the effect of a whole-gene deletion were the same as a loss-of-function point variant, then, due to the 1 in 3–6,000 frequency of 22q11.2DS, we should expect to find one 22q11.2 deletion for every 12 to 25 *LZTR1*-associated schwannomatosis diagnoses. Therefore, in our cohort of 247 *LZTR1*-associated schwannomatosis patients, we should have found a minimum of ten 22q11.2 deletions, but none were found.

## DISCUSSION

In the general population, 1 in 500 people will develop a sporadic schwannoma (1 in 1,000 will develop a vestibular schwannoma and 1 in 1,000 will develop a nonvestibular schwannoma).^9^ While the majority of heritable predisposition to schwannomas is known to be associated with *NF2* germline variants, a proportion of these have also been associated with *LZTR1* variants.^1,2^ In our review of 1,556 people with proven 22q11.2DS (89% with *LZTR1* deletion) none had a schwannoma. This finding was confirmed by brain MRI in 418 people, and by spine MRI in 94 people, in this group. Assuming a similar proportion in the self-reported unconfirmed UK based survey of 22q11.2DS, a further 1,508 are likely also to have had an *LZTR1* deletion. This would mean that close to 3,000 people with *LZTR1*-deleted 22q11.2DS were included in our analysis. Although many of these patients are still young, a large number would have been expected to have developed schwannomas if the risks were similar to typical *LZTR1* loss-of-function variants, since the median age at onset in people with a pathogenic *LZTR1* variant is 38 years in our cohort and 20% developed symptomatic tumors below the age of 29 (~85% of the 22q11.2DS cohort is below this age). It would also be reasonable to expect to have seen at least one by chance, i.e., population risk. However, life expectancy is reduced in 22q11.2DS and the presence of acute and life-threatening complications (such as CHD, hypoparathyroidism, psychosis, cervical spine anomaly) limits our current assessment.

The absence of reported schwannoma in the literature and in two large series of patients with 22q11.2DS suggests that the typical large deletion at 22q11.2, including *LZTR1*, is not a risk factor for schwannoma. While whole-gene deletions and single and multiple exon deletions occur as a relatively frequent pathogenic variant type in tumor predisposition syndromes, whole-gene deletions are not normally associated with loss of heterozygosity (LOH) as the second loss-of-function event for that gene.^10^ This does not negate the potential for biallelic loss of function; however, the second hit is nearly always a single-nucleotide pathogenic variant in the wild type allele. In the case of *LZTR1*, a single-nucleotide pathogenic variant as a second hit would not lead to loss of *NF2* on the same allele and would almost certainly not advance schwannoma formation. Alu repeats in the deleted region lead to limited mechanisms of loss by recombination on that allele, leaving only the option of biallelic *NF2* single-nucleotide pathogenic variants, which occur as the cause in <30% of sporadic vestibular schwannomas with both hits identified.^11^ It is possible that this could have occurred in the single case we observed with a large 22q11.2 deletion and a schwannoma, or the tumor could have occurred as a chance association through biallelic single-nucleotide pathogenic variants within the *NF2* gene_._

Assuming that somatic loss of the normal copy of 22q is mechanistically still possible in patients with 22q11.2DS, it is indeed possible that homozygous deletion of such a large region, involving multiple genes, might be cell lethal, thus preventing tumor development. Some support for this theory comes from analysis of copy-number alterations found in cancer cell lines, e.g., using the copy-number analysis (CONAN) database (http://www.sanger.ac.uk/cgi-bin/genetics/CGP/conan/search.cgi) available through the Wellcome Trust Sanger Institute Cancer Genome Project (accessed 11 June 2020).^11^ It also remains possible that a complete absence of LZTR1 protein due to deletion does not provide the same pathogenic effect as a partially functional protein.^12^ In addition, the normal mechanism of sporadic schwannoma formation with a point variant and LOH of *NF2* would only be possible if the single-nucleotide pathogenic variant occurred in *trans* with the 22q11.2 deletion, further reducing the chances of even a sporadic schwannoma. It is therefore possible that if loss of both copies of the whole wild type 22q11.2 allele is cell lethal, then 22q11.2 deletion syndrome patients may have a reduced risk of schwannoma as well as other 22q mediated tumors such as meningioma. However, only further follow up of an even larger series of patients with a proven chromosome 22q11.2 deletion inclusive of *LZTR1* into older age will assess the validity of this possibility.

## Data Availability

Anonymized research data are available upon request.
